# Rab23’s genetic structure, function and related diseases: a review

**DOI:** 10.1042/BSR20160410

**Published:** 2017-03-02

**Authors:** Li-Qiang Zheng, Su-Min Chi, Cheng-Xin Li

**Affiliations:** 1 Department of Dermatology, Chinese PLA General Hospital, Beijing, China; 2 Department of Dermatology, the 251st Hospital of Chinese PLA, No.13.Jian'guo Road, Zhangjiakou City, Hebei Province, 075100, China; 3 Department of Physiology, Fourth Military Medical University, Xi'an, China

**Keywords:** function, gene, G-proteins, Rab23

## Abstract

Rab23 has been proven to play a role in membrane trafficking and protein transport in eukaryotic cells. Rab23 is also a negative regulator of the Sonic hedgehog (Shh) signaling pathway in an indirect way. The nonsense mutation and loss of protein of Rab23 has been associated with neural tube defect in mice and aberrant expression in various diseases in human such as neural system, breast, visceral, and cutaneous tumor. In addition, Rab23 may play joint roles in autophagosome formation during anti-infection process against Group A streptococcus. In this review, we give a brief review on the functions of Rab23, summarize the involvement of Rab23 in genetic research, membrane trafficking, and potential autophagy pathway, especially focus on tumor promotion, disease pathogenesis, and discuss the possible underlying mechanisms that are regulated by Rab23.

## Introduction

Rab proteins belong to the Ras small GTPases superfamily. Up to date, more than 70 members have been identified in mammals. Each of them is specifically localized to the cytoplasmic surface of a distinct membrane-bound organelle and appears to regulate the intracellular traffic [1–3]. Rab proteins cycle between a cytosolic GDP-bound off-state and a membrane-associated GTP-bound on-state, which are orchestrated by three types of upstream regulators, namely, GDI (GDP dissociation inhibitor), guanine nucleotide exchange factor, and GTPase activating proteins. Different active form of Rab proteins interacts with downstream effectors to exert its biological functions [4–6].

It has been indicated that Rab proteins regulate not only membrane trafficking or autophagic pathway [4], but also cell signal transduction [7], survival, and development [8,9]. Abnormal expression of Rab proteins and their regulators or effectors are closely related to many diseases, such as cancer, neuropathy, cardiovascular, Type 2 diabetes, and lipid metabolism disorders [5,6,10].

Rab23 is one of Rab proteins that have been explored extensively [11]. During the last three decades, at least two studies have revealed that Rab23 may play an important role in endosomal membrane trafficking [12,13]. Also, it negatively regulates Sonic hedgehog (Shh) signaling pathway in an indirect way [14]. Moreover, genetic null mutation or trafficking disorders of Rab23 can lead to human diseases including carpenter syndrome (CS) [15–18], malignant tumor [19–27], susceptible to pathogen infection [28,29], and other related diseases [30,31].

In the following sections, we gave an overreview on the functions of Rab23, summarized the involvement of Rab23 in genetic research, intracellular trafficking, potential autophagy process, tumor promotion, disease pathogenesis, and explored the possible underlying mechanisms that are regulated by Rab23. The role of miRNAs in regulating Rab23 expression epigenetically was also discussed.

## *Rab23* gene and its mutation study

In 1994, employing rapid amplification of cDNA ends techniques, Olkkonen et al. [32] reported the cloning of a full-length cDNA encoding mouse Rab23. Surprisingly, the Rab23 amino acid sequence is not more tightly associated with any previously documented small GTPase, but belongs to the Rab subfamily [32]. Moreover, Rab23 mRNA is mainly expressed in the brain, similar to Rab3a and Rab15, suggesting that Rab23 has a function in a process of brain development, and that the modulation of transcription or splicing of *Rab23* gene in the brain may be distinguished from other tissues [32].

Subsequently, there have been increasing number of studies focusing on genetic function of Rab23. In mammals, Gunther et al. [33] identified the mouse *open brain *(*opb*) gene based on two recessive mutations (spontaneous and induced by N-ethyl-N-nitrosourea respectively). Next, homozygous *opb* embryos showed an exencephalic monstrosity affecting the forebrain, midbrain, and hindbrain regions [33]. However, the definite relationship between mouse *opb* gene and Rab23 has remained unclear. Until 2001, Eggenschwiler et al. [14] cloned *opb* gene and found that it encodes Rab23, which demonstrates that dorsalizing signals activate transcription of Rab23 in order to antagonize the Shh pathway in dorsal neural cells. Namely, *opb* and *Shh* genes have opposing roles in neural patterning: *opb* is required for dorsal cell types, whereas *Shh* is required for ventral cell types in the spinal cord. Furthermore, researchers also found that *opb* acts downstream of *Shh* [14].

Further study in mouse revealed that Rab23 protein is highly enriched in the adult brain and present in low levels in other tissues [13,34]. In the adult mouse brain, Rab23 expression is found most prominently in the cortex, hypothalamus, and the cerebellum. However, it is absent from astrocytes (positive marker for glial fibrillary acidic protein) or oligodendrocytes (positive for CNPase) [13,15]. In thalamic neurons, overexpression of Rab23 or its mutants also does not affect the morphological differentiation *in vitro*. Surprisingly, recessive embryonic lethality with neural tube defects is caused by nonsense mutations in Rab23 in *opb* mice, suggesting a species difference in the requirement for Rab23 during embryo development. The exact mechanism of different function of the Rab23 protein in different development stage has been completely unknown [13].

Although Rab23 defect can cause human CS in autosomal recessive inheritance mode, the dominance of loss-of-function mutations in human probands leads to a CS phenotype without neural tube defects or lethality [18]. This disease is a rare acrocephalopolysyndactyly (OMIM201000) characterized by acrocephaly, brachydactyly, and syndactyly in the hands, and preaxial polydactyly and syndactyly in the toes with markedly intrafamilial variability [35]. Generally speaking, incorrect embryogenesis underlying CS is believed to occur between the embryo 30 and 49 days.

Using homozygosity mapping, Jenkins et al. [15] identified the gene of this syndrome is loctated at chromosome 6p12.1-q12 and simultaneously reported five gene mutations. This gene comprises one noncoding and six coding exons, with an interval region of 35.43 kb. For example, nonsense mutation of homozygosity, L145X, causing ten patients with CS, which locates on a common haplotype and indicates a founder effect in patients of Northern European descent [15]. The discovery of Rab23 mutations in CS also implicates Shh signaling in cranial suture biogenesis and provides a new molecular target for studies of obesity. Subsequently, investigation by Ben-Salem et al. [17] demonstrated a novel splicing mutation (c.482-1G>A) of *Rab23* gene in a consanguineous Emirati family. This mutation eliminates the acceptor splice site of exon 5, which results in deleting 8 nt in the Rab23 mRNA by a frameshift and premature termination codon at position 161 (p.V161fsX3) [17].

More recently, Haye et al. [36] reported a patient of CS with another novel Rab23 mutation with nonspecific ultrasound findings of abnormal skull shape, complicate cardiac defect, cystic hygroma, and bowed femora on the prenatal findings in a fetus. Moreover, retrospective detection on fetal CT scan found craniosynostosis and preaxial hexadactyly of the feet. Finally, the same homozygous mutation leading to a skip of exon 6 and premature termination codon (c.481G>C; p.Val161Leufs16) was identified [36]. Therefore, prenatal imaging in cases of abnormal skull shape associated with bowed femora and/or heart defect provides another evidence for the diagnosis of CS.

Another Rab23 mutation (c.86dupA) was reported in the homozygous state in four relatives of Comorian origin [16]. All patients presented with acrocephaly and polysyndactyly. However, the variability within kindred is observed with different severity of craniosynostosis ranging from cloverleaf skull to mainly involving metopic ridge. All children also showed a combination of brachydactyly with agenesis of the middle phalanges and variable polydactyly or syndactyly in the hands and toes [16]. Evaluation of mental development is normal in all four patients except that the eldest one presented with impaired motor development due to orthopedic complications [16]. Cranial-computed tomography revealed hydrocephalus with additional abnormal clinical features.

So far, at least 12 *Rab23* gene mutations have been documented and further job needed to be done to identify more mutation sites [17,37]. Collectively, the diverse phenotype observed in CS has established that Rab23 mutation has a role in a range of phenotypic changes, including cranial suture formation and growth, central nervous system and acral development, fat metabolism and left–right lateralization etc [18]. Generally speaking, these developmental defects in CS resulting from Rab23 nonsense mutations are largely attributed to aberrant signaling from the cilia, and a big part of this may be Shh signaling; while nodal signaling only accounts for a little part.

## Rab23’s role in plasma membrane recycling and transport

However, expression of Rab23 in the adult brain suggests that it has a post-natal function beyond its role in embryonic development. In recent years, tremendous progress has been made in understanding the roles of Rab23 during various steps of membrane trafficking.

Evans et al. [12] analyzed the localization of Rab23 by light and immunoelectron microscopy after stable expression of wild-type (Rab23wt), constitutively active Rab23 (Rab23Q68L), and inactive Rab23 (Rab23S23N) in a range of mammalian cell types. Rab23wt and Rab23Q68L are predominantly localized to the plasma membrane but are also linked to intracellular vesicular structures, whereas Rab23S23N is primarily cytosolic. Vesicular Rab23wt may colocalize with Rab5Q79L and internalized transferrin-biotin, but not with the late endosome maker LBPA or the Golgi complex maker GM130 [12]. To further investigate the role of Rab23 in Shh signaling pathway, Rab23wt was co-expressed with either patched or smoothened. Consequently, patched colocalizes with intracellular Rab23wt but smoothened does not [12]. Analysis of patched distribution revealed that it is predominantly localized to endosomal elements, including early endosomes with transferrin receptor-positive expression and putative endosome carrier vesicles, rather than plasma membrane [12]. Rab23wt or Rab23 mutant does not alter the distribution of patched or smoothened, suggesting that it is likely that Rab23 acts more distally in regulating Shh signaling in spite of the endosomal colocalization of Rab23 and patched. So, detailed analyses at both the genetic and cellular levels will make Rab23 placed in the context of the molecular hierarchy of Shh signaling, contributing to understanding Rab23-mediated trafficking events.

By detecting interaction between Rab23 and Shh signaling molecules including smoothened, Su(Fu), and Gli1, our research group found that Rab23wt can suppress Gli1 transcriptional activity but not in Su(Fu) null fibroblasts [38], using Gli1-mediated reporter gene analysis. Similarly, Rab23wt expression reduces the nuclear localization of Gli1 but not Su(Fu) null fibroblast cells. Consistent with the GTPase motif in the protein, we also identified that Rab23 has GTPase activity. Rab23S23N is unable to suppress Gli1-mediated transcriptional activity [38]. Taken together, these data provide evidence to support that Rab23 negatively regulates Gli1 activity in a Su(Fu)-dependent manner.

The structure and function of the primary cilium as a sensory organelle is dependent on a motor-protein-powered intraflagellar transport system. Based on fluorescence recovery after photo-bleaching (FRAP), Boehlke et al. [39] confirmed that Rab23 shRNA or Rab23S23N decreases the cilium (renal epithelial-derived MDCK cells) steady state exclusively for smoothened but not for EB1 (also known as microtubule-associated protein RP/EB family member 1) or Kim1, suggesting a role of Rab23 in protein turnover in the cilium.

To test Rab23’s role in the ciliary targeting of known cargoes, Lim and Tang [40] found in Rab23 silenced cells, ciliary localization of a kinesin KIF17 is disrupted, which suggests that Rab23 has a novel role in controling KIF17’s ciliary transport. Appropriate physiological signaling by primary cilia also depends on the specific targeting of particular receptors to the ciliary membrane. More recently, the observation by Leaf and Von Zastrow [41] demonstrated D1-type dopaminergic receptors are delivered to cilia from the extra-ciliary plasma membrane by a mechanism requiring the receptor cytoplasmic tail, the intraflagellar transport complex-B (IFT-B), and ciliary motor KIF17. This targeting mechanism critically relies on Rab23. Dopamine receptors are prevented from entering the cilia by reducing the amount of Rab23 in a cell. Conversely, fusion of Rab23 with a non-ciliary receptor is enough to drive robust, nucleotide-dependent mis-localization to the ciliary membrane. Finally, Leaf and Von Zastrow [41] raised four interesting questions for future study: (a) How to control Rab23 nucleotide state? (b) How the mechanism of selective cargo communication with the ciliary delivery is determined? (c) How receptor is directed by Rab23 delivery to the ciliary membrane? (d) What broader physiological roles the discrete Rab23-dependent ciliary targeting mechanism has? Thus, further investigation of the receptor-specific ciliary targeting mechanism may provide fundamental insight into the role of primary cilia and toward understanding pathologies linked to ciliary defects.

Asymmetric fluid flow in Nodal signaling in the left lateral plate mesoderm (LPM) contributes to the mammalian left–right patterning. Rab23 is not required for initial symmetry breaking in the node. However, it is needed for expression of Nodal and Nodal target genes in the LPM. Microinjection of Nodal protein or transfection of Nodal cDNA in the embryo revealed that Rab23 is required for the production of functional Nodal signals, rather than the response to them [42]. Using gain- and loss-of function approaches, research group showed that Rab23 plays a similar role in zebrafish, where it is required in the teleost equivalent of the mouse node, Kupffer’s vesicle. Collectively, these data suggest that Rab23 is an essential member of the mechanism that transmits asymmetric patterning information from the node to the LPM independent of the Shh pathway [42].

As described above, myriad functions of Rab23 have surpassed embryonic developmental stages and Shh signaling. Therefore, further investigation to construct conditional transgenic or knockout animals make it more easily subjected to selective spatial and temporal control to analyze expression and function of Rab23 in the brain.The cellular and physiological phenotype that is linked to Rab23 might then be clarified in more detail [43].

## Rab23’s functions in antibacterial defense and potential role in autophagy

Autophagy (here means macroautophagy) is a highly conserved intracellular and lysosome-dependent degradation process in which autophagic substrates are enclosed and degraded by a double-membrane vesicular structure in a continuous and dynamic vesicle transport process [4]. Increasing evidence has demonstrated that many Rab proteins regulate the vesicle traffic processes [1]. Furthermore, there are several overlaps between endocytosis and autophagocytosis observed in mammalian cells due to the localization of endocytosed material in autophagic vacuoles.

To date, three types of mechanism of Rab proteins involving the autophagy process have been reported. One is participating in autophagic membrane formation [44], the other is in association with autophagy signal transduction pathway [45]. The last mechanism involves both of them [46].

Considerable Rab proteins have been shown to be linked to various stages of autophagy. The observation by Smith et al. [29] revealed the association of 48 Rab proteins with model phagosomes including a non-invasive mutant of Salmonella enterica serovar Typhimurium (S. Typhimurium). This mutant traffics to lysosomes may decide which Rab protein localizes to a mature phagosome. Consequently, 18 Rab proteins are related to maturing phagosomes, each of them with different kinetics of association. Rab23S23N and Rab35S22N both inhibit phagosome–lysosome fusion [29]. Moreover, a number of Rab GTPases localizing with wild-type Salmonella-containing vacuoles (SCVs) do not fuse with lysosomes. However, some Rabs (5, 7A, 11A, and 11B) are recruited to wild-type SCVs whereas others (8B, 13, 23, 32, and 35) are excluded from this compartment [29]. These results demonstrated that a complicate network of Rab GTPases controls endocytic trafficking to lysosomes and that this is regulated by S. Typhimurium to allow its intracellular growth. Future studies are required to clarify the molecular mechanisms of Rab GTPases controling phagosome maturation, and how intracellular pathogens like S. Typhimurium regulate their function.

Since Rab23 plays a role in the anti-pathogens activity of the endogenous autophagy machinery [29], it is biologically plausible that dysfunction might result in decreasing autophagic clearance after the lung epithelium was exposed to bacterial pathogens. Overexpression of Rab23 not only in the lung but also in the small intestine and colonic mucosa may hint a broader potential role of Rab23 for antibacterial defense mechanisms in epithelial barrier organs. Quantitative analysis of Rab23 mRNA indicated the gene is one of the most likely risk factors. The result defined that Rab23 takes part in antibacterial defense processes. As mentioned above, the gene locus of Rab23 localizes to chromosome 6p12.1-q12. The identified association of the 6p12.1 locus with sarcoidosis further implicates this locus may be a crossing susceptibility factor and Rab23 may play a potential role in the pathophysiology of sarcoidosis [47].

Later, the finding by Nozawa et al. [28] identified Rab9A and Rab23 as a novel autophagy regulating partner against Group A streptococcus (GAS) infection. Rab9A is recruited to GAS-containing autophagosome-like vacuoles (GcAVs) after autophagosomal maturation and its activity contributes to GcAV enlargement and eventual fusion with lysosome. GcAV enlargement seems to be linked to homotypic fusion of GcAVs with Rab9A. Rab23 is recruited to GAS-capturing forming autophagosomes. Knockdown of Rab23 expression decreases the number of autophagosomes containing GAS [28]. It is therefore suggested that Rab23 is required for GcAV formation and is involved in GAS targeting of autophagic vacuoles. Furthermore, knockdown of Rab9A or Rab23 expression impairs the degradation of intracellular GAS. Therefore, these data revealed that the Rab9A and Rab23 GTPases play crucial roles in autophagy of GAS. However, neither Rab9A nor Rab23 are co-localized to nurtirent-deprived autophagosomes. Although both Rab9A and Rab23 are dispensable for starvation-induced autophagosome formation [28]. These results indicate Rab23 is localized in forming autophagosomes, autophagosomes as well as to autolysosomes and vanish from GcAVs during the later stages of autophagy. However, the Streptococcus-containing and digesting vacuoles described by Nozawa et al. may be autophagosome-like, but whether these are truly autophagosome need to be proven[28].

Similar to Rab23, Oda et al. [48] identified Rab30 as a more novel regulator of GcAVs. Rab30, a Golgi-resident Rab, is recruited to GcAVs in response to autophagy induction by GAS infection in epithelial cells. The difference is that Rab30 colocalized with a starvation-induced autophagosome. These results suggest that Rab30 mediates autophagy against GAS independently of its normal cellular role in the structural maintenance of the Golgi apparatus.

As an outer stress factor, UV can induce cell autophagy. To evaluate the role of Rab23 in UVB-induced autophagy, our research group established stably Rab23 expressing and Rab23 knocking down HaCaT cells respectively. The expression of Rab23 is verified by immunoblotting. Upon 30mJ/cm2 UVB exposure, Rab23wt increases the expression of LC3-II and Beclin1, while Rab23 RNAi decreases the expression of LC3-II and Beclin 1. Consistent with this result, the finding of fluorescence microscopy revealed that Rab23wt significantly increases the number of autophagosome (unpublished data, private communication, C. Lin).

To date, all experiments only implicate that Rab23 play a potential role in autophagy process, such as forming autophagosome-like vacuoles or increasing expression of autophagy flux makers, no finding about its linking to autophagy signal pathway. More convincing evidence directly to prove the role of Rab23 during autophagy is sure to a new research target.

## Rab23’s functions in tumor

Rab GTPases are disregulated in many cancers and play a multitude of roles in tumor cell proliferation, migration, invasion, communication with stromal cells, and the development of drug resistance [10].

As to Rab23, there exists some evidence that it plays a negative regulator in some carcinogenesis. As previous documents reported, Rab23 acts as a negative regulator of Shh signaling in vertebrate neural systems. Aberrant activation of this signal pathway in post-natal life has been associated with a range of cancers including lung, prostate, colorectal, and cutaneous basal cell carcinomas. Therefore, the absence of the negative regulator of Shh signaling, Rab23, may be implicated in the development of thyroid carcinoma. As expected, Denning et al. [49] found that down-regulation of Rab23 is seen in three malignant cohorts: follicular thyroid carcinoma, papillary thyroid carcinoma, and follicular variant of papillary thyroid carcinoma when compared with the benign follicular adenoma. Again, this result demonstrates Rab23 is a tumor-suppressing gene.

However, more and more accumulative data suggest that Rab23 as an oncogene (Table 1). Overexpression of Rab23 not only is found in precancerous lesion like atrophic gastritis with intestinal metaplasia [50] and actinic keratosis [27], but also has been implicated in several human cancers. Liu et al. [19] firstly elucidated the role of Rab23 in hepatocellular carcinoma (HCC) by evaluating the expression of Rab23 in HCC tissue and in HCC cell lines. High cytoplasmic and nuclear expression of Rab23 is found in 53.5 and 72% HCC patients respectively, which correlated with tumor size. Survival rates at 24 and 48 h for Hep-3B cells transfected with siRNA for Rab23 are lower and approximately 30% Hep-3B cells were apoptotic. Knocking down rab23 suppresses Hep3B cell growth, suggesting that overexpression of Rab23 play a key role in Hep3B cell growth. Later, Sun et al. [51] further investigated the role of Rab23 in HCC and reported the distinct sublocalization pattern of Rab23 in HCC cell lines (nuclear expression in HepG2, not Hep3B). This difference relies on the GDP/GTP-binding form, and inhibition of the Rab23 cycle decreases the expression and nuclear localization of Gli1. In consistence with this, Huang et al. [21] also detected the expression of Rab23 protein in the nuclei in lung cancer tissues by immunohistochemistry. In this context, Rab23 may play a more direct role in Gli-mediated transcription.

**Table 1:** Rab23’s regulatory roles in tumorigenesis and invasion

Authors	Year	Type of tumor	*In vitro* or *in vivo*	Expression level and positive location
Liu, Y.J. et al. [19]	2007	Hepatocellular carcinoma	Tissue and cell line	Higher protein in nucleus
Hou, Q. et al. [20]	2008	Gastric cancer	Tissue and cell line	Higher mRNA and protein in cytoplasma
Zeng, C. et al. [56]	2009	Breast cancer	Cell line	Higher protein in cytoplasma
Zeng, C. et al. [54]	2010	Breast cancer	Cell line	Higher mRNA and protein in cytoplasma
Huang, S. et al. [21]	2011	Lung cancer	Tissue and cell line	Higher mRNA and protein in nucleus
Sun, H.J. et al. [51]	2012	Hepatocellular carcinoma	Cell line	Higher protein in nucleus
Zhao, J. et al. [55]	2013	Breast cancer	Cell line	Higher protein in cytoplasma
Zhang, H.H. et al. [22]	2013	Non-small cell lung cancer	Cell line	Higher mRNA and protein (unknown location)
Cai, Z.Z. et al. [63]	2015	Pancreatic duct adenocarcinoma	Tissue and cell line	Higher mRNA and protein in cytoplasma
Wei, C.J. et al. [23]	2015	Gliomas	Tissue and cell line	Higher mRNA and protein in cytoplasma
Liu, Y. et al. [24]	2015	Breast cancer	Cell line	Higher mRNA and protein in cytoplasma
Wang, M. et al. [25]	2016	Astrocytoma	Tissue and cell line	Higher mRNA and protein in cytoplasma
Jiang, Y. et al. [26]	2016	Bladder cancer	Tissue and cell line	Higher protein in cytoplasma
Jian, Q. et al. [27]	2016	Cutaneous squamous cell carcinoma	Tissue and cell line	Higher mRNA and protein in cytoplasma
Cheng, L. et al. [53]	2016	Esophageal squamous cell carcinoma	Tissue and cell line	Higher mRNA and protein in cytoplasma

Unlike in HCC and lung cancer, the localization of Rab23 in other cancer tissue or cell lines is cytoplasmic positivity. Hou et al. [20] identified Rab23 as an amplified and overexpressed Chr 6p11p12 gene in Hs746T cells. siRNA silencing of Rab23 significantly reduces cellular invasion and migration in Hs746T cells, whereas overexpression of Rab23 enhances cellular invasion in AGS cells. Moreover, Rab23 expression is significantly associated with diffuse-type GC compared with intestinal-type GC [20]. Our research group found that the expression of Rab23 mRNA is higher in MDA–MB-231 and Bcap-37 than MCF-7 and a normal breast cell line HBL-100 [54]. Bcap-37 cell line with Rab23 overexpression has dramatically enhanced migration and invasion potentials, while Rab23 knockout shows the opposite effects [55]. However, relevant experiments demonstrated that overexpression of Rab23 protein may cause cell cycle arrest in the G1 phase and a decrease in the S phase population as well as induction of apoptosis. Furthermore, Rab23 decreased Gli1 and Gli2 mRNA levels when compared with the control group. Therefore, our results indicated that ectopic expression of Rab23 inhibits the growth and proliferation independent of breast cancer’s estrogen receptor, as well as induces cell apoptosis in breast cancer cells [24,56]. These effects may be due to the inhibition by Rab23 of Gli1 and Gli2 mRNA expression. Hence, further research is needed to confirm this varying quantities [24].

With regard to cutaneous squamous cell carcinoma, our research group found that the expression level of Rab23 is higher in moderately to poorly tumor differentiation tissue and non-exposed sites [26,27,57]. Interestingly, Rab23 RNAi can suppress cell invasion and Rab23 overexpression promotes cell invasion depending on GTP-bound form of Rab23 [27,58]. Moreover, Rab23Q68L facilitates oncogenesis in nude mice while Rab23S23N restrains tumor development.

As a member of the Rac subfamily of the Rho family GTPases, Rac1 functions as a pleiotropic regulator of many cellular processes involving cell cycle, adhesion, motility, and epithelial differentiation [59,60]. Abnormal Rac1 activation has been found in several cancers and promotes cancer cell motility, invasion, and metastasis [61]. Inhibition of Rac1 activity or Rac1 siRNA attenuates Rab23 enhancing cells migration and invasion [62]. Notably, our research group confirmed that Rab23 is co-localized with integrin β1 in cell membrane of Rab23wt and Rab23Q68L stable expression cells and Rab23 efficiently co-precipitates with integrin β1 and Tiam1 in a GTP-dependent manner. Further, integrin β1 siRNA interruptes the co-precipitation between Rab23 and Tiam1 and attenuates the role of Rab23. Taken together, our results indicated that Rab23 promotes squamous cell carcinoma cells migration and invasion by regulating Integrin β1/Tiam1/Rac1 pathway [27]. Similarly, Rab23 serves as an important oncoprotein in human astrocytoma by regulating cell invasion and migration through Rac1 activity. Handling of transfected cells with Rac1 inhibitor (NSC23766) decreases Rac1 activity and invasion [25]. However, Rac1 is not the downstream effector of Rab23.

## Who regulates Rab23?

Changes in Rab23 levels in normal and cancer tissues could be regulated by both genetic and epigenetic factors [63]. To date, there are some studies focusing on the role of splicing factor or miRNAs in regulating Rab23 in transcriptional or post-transcriptional level.

Binding of PSF (proline-and glutamine-rich splicing factor) to Rab23 DNA were analyzed by Wang et al. [64] by ChIP technology in both NIH/3T3 and B16F10 cell lines with the result of PSF binding and inhibiting transcription of the Rab23 gene. In addition, knocking down Lime23 (long non-coding RNAs) obviously represses the malignant property of YUSAC melanoma *in vitro*, accompanied by the suppression of proto-oncogene Rab23 [65]. Abnormal expression of miRNAs is often found in some cancers, and it promotes the pathogenesis of cancer via regulation of the cell proliferation, apoptosis, migration, and invasion. miR-200b, as a tumor suppressor in multiple tumors including glioma, breast cancer, gastric cancer, and ovarian cancer, is down-regulated in glioma tissues and its lower expression is associated with poor prognosis [66]. Members of Rab family, Rab21, Rab23, Rab18, and Rab3B are predicted to be novel targets of miR-200b. The patients who show the up-regulation of all the four Rabs have the worst outcome, vice versa [66,67]. Likewise, the expressions of the four Rabs are repressed by transfection of miR-200b in breast cancer cells. Overexpression of miR-200b or knockdown of Rab21, Rab23, Rab18, and Rab3B inhibits breast cancer cell proliferation and invasion *in vitro*. These data provide evidence that miR-200b is a prognostic factor in breast cancer targeting multiple members of Rab family [66]. Recently, ectopic expression of Rab23 could reverse the migration and invasion inhibitory activity of miR-367 suggesting that Rab23 is also a target gene of miR-367 [68,69]. In the further study, Cheng et al. [53] found that the expression of Rab23 is regulated by the miR-92b. Forced expression of miR-92b decreased the mRNA and protein level of Rab23, and Rab23 rescued the biological functions of miR-92b. The level of Rab23 was significantly increased in the pain rat model, and intrathecal injection of Rab23 aggravated pain response. Furthermore, Rab23 was negatively regulated by miR-16 and also confirmed as a direct target of miR-16 [70].

## Other diseases caused by dysfunction of Rab23

In certain pathological states, both Rab-family and hedgehog-related proteins are involved in sclerosis and fibrosis. Many members of the Rab family, such as Rabs 3A, 5, 13, 18, 25, and 38, have been identified in the kidney [71]. Huang et al. [30] confirmed that overexpression of Rab23 is observed in the urine of the focal segmental glomerulosclerosis (FSGS) mice, but not in the serum. Rab23 and Shh signaling pathway genes are constitutively expressed in normal kidneys and remarkably up-regulated in the kidneys of FSGS mice. The basal expression of Rab23 is identified in glomeruli, and mesangial cells (MCs) show prominent elevation of Rab23 in the FSGS state. The gain and loss of Rab23 function can cause up-regulation and down-regulation of collagen typeI A2 respectively, whereas the expressions of fibronectin and other types of collagen are not affected [30]. This result indicated that Rab23 contributes to FSGS by affecting the synthesis of certain extracellular matrix proteins. An autocrine loop of hedgehog signaling could be activated in MCs in the FSGS state, and could serve as a biomarker that indicates the severity of FSGS [30].

Subsequently, Huang et al. [31] investigated the effects of different levels of Rab23 activity (including Rab23shRNA, Rab23wt, Rab23Q68L, and Rab23S23N) on proteome of various biological pathways. Proteomic analysis revealed the potential roles for Rab23 in multiple processes, including signal transduction, transcription modulation, protein synthesis and degradation, cytoskeleton reorganization, circadian rhythm regulation etc [31]. Bioinformatics analyses showed that Rab23 has an impact on multiple biological networks in MCs. These data may elucidate the roles of Rab23 in mesangiopathy or MC damage.

In addition, during the course of chondrocyte development, Yang et al. [72] found that up-regulation of Rab23 inhibits chondrogenic differentiation with a simultaneous lower expression of matrix protein such as collagen type II and aggrecan. Moreover, Rab23 siRNA also results in inhibition of chondrogenic differentiation as well as down-regulation of Sox9 (a master regulator of chondrogenesis). Interestingly, Sox9 expression has recently been linked to Gli1. And Rab23 knockdown decreases Gli1 expression in chondrocytes [72]. Because the phenotypes of Rab23 mutations in mice and humans include defects in cartilage and bone development, the present study suggests that Rab23 regulates Sox9 expression via Gli1 protein.

## Perspectives

**Figure 1:**
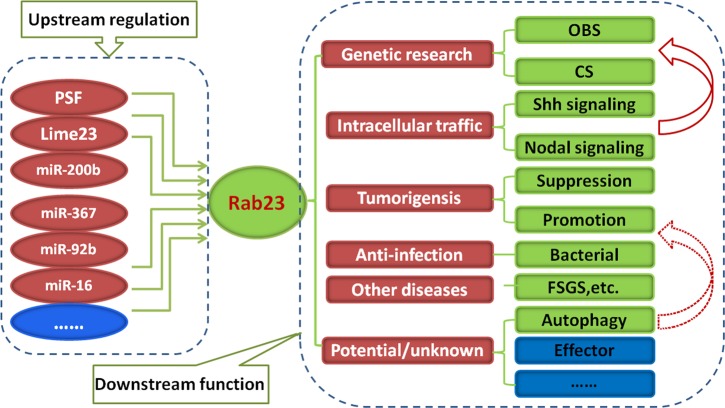
A simplified schematic diagram illustrates that Rab23 is regulated epigenetically by some molecules in the upstream, such as known or unknown small RNAs Likewise in the downstream, some important roles have been analyzed in the text, with a focus on genetic research, intracellular traffic, and tumorigensis. Firstly, the mutation of Rab23 in mouse may cause *opb* Syndrome, while in human for CS. These developmental defects in CS are largely down to aberrant signaling from the cilia, and a big part of this may be Shh signaling (solid arrow), a little part for nodal signaling. Secondly, the function of Rab23 in tumor resembles a ‘double sword’, indicating promoting or inhibiting tumorigensis. Moreover, potential or unknown role in autophagic process has been discussed, which may be linked to anti-bacterial infection or tumorigensis (dotted arrow). Finally, effector of Rab23 is also a research direction in the future.

In conclusion, Rab23 is a vital regulatory molecule with multiple capacity of regulating endocytic pathway, Shh signaling, tumor invasion, and metastasis, but potentially essential roles in autophagy, development, and other diseases have yet undefined (Figure 1). Whether Rab23 acts as an oncogenic protein or a tumor suppressor is likely cellular context dependent. Most important, Rab23’s effector has remained unclear. Also, there have not been a better animal model, like transgenic mice, focusing on the research of function of Rab23 gene. Based on the uncommon characteristics of Rab23, in the future it will bring significant interest and challenges in developing therapeutic methods from gene therapy to small molecule interventions.

## Abbreviations

CS: carpenter syndrome
EB1: end-binding protein1
ECM: extracellular matrix
ESCC: esophageal squamous cell carcinoma
FGFR3: fibroblast growth factor receptor 3
FRAP: fluorescence recovery after photo-bleaching
FSGS: focal segmental glomerulosclerosis
GAS: Group A streptococcus
GcAVs: GAS-containing autophagosome-like vacuoles
GDF: GDI displacement factor
GDI: GDP dissociation inhibitor
GEF: guanine-nucleotide-exchange factor
HCC: hepatocellular carcinoma
IFT-B: intraflagellar transport complex-B
KIF17: kinesin family member 17
LBPA: lysobisphosphatidic acid
LC3-I/II: microtubule associated protein 1 light chain 3 I/II
LPM: lateral plate mesoderm
MC: mesangial cell
MDCK: Madin Darby Canine Kidney cell
opb: open brain
PDAC: pancreatic ductal adenocarcinoma
PSF: proline-and glutamine-rich splicing factor
Rab23Q68L: constitutively active Rab23
Rab23S23N: inactive Rab23
Rab23wt: Rab23 wild-type
Rab5Q79L: constitutively active Rab5
SCV: Salmonella-containing vacuole
Shh: Sonic hedgehog
shRNA: short hairpin RNA
Sox9: SRY-related high mobility group-box gene9
UVB: ultraviolet
